# A journey through the anatomy of the cerebellum

**DOI:** 10.1055/s-0045-1814379

**Published:** 2025-12-22

**Authors:** João Vitor Gerdulli Tamanini, Raphael Pinheiro Camurugy da Hora, Luís Gustavo Biondi Soares, Luiz Fernando Monte Borella, Fabiano Reis, Luis Ángel Canache, Victor Rebelo Procaci, Feres Chaddad-Neto, Flávio Moura de Rezende Filho, Orlando Graziani Povoas Barsottini, José Luiz Pedroso

**Affiliations:** 1 Universidade Federal de São Paulo, Escola Paulista de Medicina, Departamento de Neurologia e Neurocirurgia, São Paulo SP, Brazil.; 2 Universidade Estadual de Campinas, Faculdade de Ciências Médicas, Departamento de Radiologia e Oncologia, Campinas SP, Brazil.; 3 Santa Casa de Montes Claros, Departamento de Neurocirurgia, Montes Claros MG, Brazil.; 4 Hospital Beneficência Portuguesa de São Paulo, Departamento de Neurocirurgia, São Paulo SP, Brazil.

**Keywords:** Cerebellum, Embryology, Radiology, Stroke, Ataxia

## Abstract

The present narrative review delves into the multifaceted roles of the cerebellum, highlighting its significance beyond traditional motor functions to encompass cognitive and behavior-related processes, as evidenced by advancements in functional neuroimaging. We provide a comprehensive summary of the cerebellum's embryological development, intricate microanatomy, macroanatomy, and vascular anatomy of the cerebellum, revealing how these aspects contribute to its unique circuitry and operational capabilities. A non-systematic literature search was conducted using the PubMed database, focusing on landmark and recent studies addressing the embryology, anatomy, clinical correlations and radiological aspects of the cerebellum. Ultimately, this review underscores the cerebellum's complex structure and diverse function, advocating for a deeper understanding to improve diagnostic and therapeutic approaches for cerebellar disorders.

## INTRODUCTION

The word *cerebellum* , derived from Latin and meaning “little brain,” was first coined in the early sixteenth century. However, despite its name, this fascinating structure is anything but little in terms of organization, circuitry and its expanding role within the nervous system, becoming a focal point in contemporary neuroscience.

Classically considered a cornerstone for motor information processing and control, the cerebellum has emerged as an important player in cognitive and behavioral functions, as demonstrated by advancements in functional neuroimaging. A deeper understanding of its extensive connectivity, both intrinsic and extrinsic, can be better achieved through a careful study of its early developmental stages and topographic organization.

Therefore, the present review aims to provide a comprehensive overview of the cerebellum's embryological, anatomical and radiological features, emphasizing its unique role in neurological function and the impact of its dysfunction in various cerebellar disorders.

## METHODS

We performed the current narrative review based on a non-systematic literature search conducted in the PubMed database up to April 30th. We included landmark studies, review articles, and recent publications related to cerebellar embryology, anatomy, function, and clinical correlations. The selection criteria prioritized relevance, scientific impact, and contribution to the anatomical and functional understanding of the cerebellum. Articles in English and Portuguese were considered, allowing the inclusion of both classical and updated references.

## THE EMBRYOLOGIC DEVELOPMENT OF THE CEREBELLUM AND CEREBELLAR PATHWAYS

The cerebellum is a crucial structure within the central nervous system, playing a vital role in the coordination of voluntary movements, maintaining balance and posture, and regulating cognitive and emotional processes. 1 Understanding the embryologic development of the cerebellum and its associated pathways is essential for comprehending the full scope of its functions and potential disruptions. 2 Moreover, the study of cerebellar embryogenesis offers insights into the complex cellular and molecular mechanisms that govern the formation of this intricate brain region, providing a foundation for understanding cerebellar function and dysfunction. 1 3

The formation of the central nervous system in the embryo begins with the neural tube. 4 5 Portions of the neural tube start secreting signaling molecules which further induce the differentiation of specific regions into particular structures. 6 In that sense, with adequate molecular stimuli, the hindbrain, or rhombencephalon, one of the primary vesicles of the neural tube, further differentiates into 7 rhombomeres. Amongst those, rhombomere 1 (r1) plays an important role as a precursor of the cerebellum.

Another important structure for cerebellar development is the isthmus, a constriction at the midbrain-hindbrain boundary positioned immediately rostral to r1. 7 This region plays a crucial role as an organizing center, orchestrating the development of the mid- and hindbrain through the secretion of signaling molecules, most notably Fgf8. 7 Specifically, Fgf8 exerts its influence by establishing gradients of morphogens, which, in turn, regulate gene expression and cell fate determination, which, ultimately, contribute to the development of r1 into the cerebellum. 6

Also derived from the r1 is the rhombic lip (RL). The RL, mostly through expression of the transcription factor Atoh1, is essential for the generation of several important glutamatergic cell populations, including the granule cell precursors of the cerebellum, as well as neurons of pontine nucleus. 6 8

Alongside the RL, the ventricular zone (VZ) is of importance for neurogenesis. The VZ, which lines the fourth ventricle and is marked by Ptf1a expression, serves as the primary source of Purkinje cells, the principal neurons of the cerebellar cortex, as well as other important neurons, such as GABAergic interneurons. 9 Those cells are also important in providing trophic support to the RL-derived cells. 10

While the VZ and the RL are generated by ventrally and intermediately located parts of the neuroepithelium, the roof plate (RP) is generated by dorsal portions of the neuroepithelium. 11 Specifically, the RP is associated with promoting signals for VZ cell proliferation and differentiation as well as the formation of the RL in itself. 12

With the intricate interactions between the RP, RL, VZ and r1 and its molecular signaling events, briefly described above, the cerebellum can successfully develop with each of its proper components. However, several abnormalities can take place in this process, leading to important cerebellar dysfunction and associated clinical manifestations. 11

**Figure 1:**
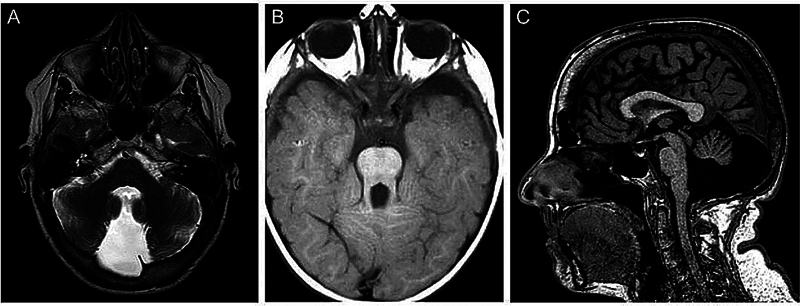
( **A** ) Axial T2 weighted magnetic resonance imaging (MRI) images of patient with vermian agenesis, and a retrocerebellar cystic structure in the posterior fossa that communicates with the fourth ventricle. ( **B** ) Axial T1-weighted MRI scan of a patient with Joubert syndrome, showing the molar tooth sign, demonstrating thickened, elongated, and parallel superior cerebellar peduncles. ( **C** ) Sagittal T1 MRI scan of a patient with Dandy-Walker syndrome, demonstrating a massive enlargement of the posterior fossa, hypoplasia and rotational displacement of the cerebellar vermis.

Amongst the cerebellar malformations, the most common one consists of the Dandy-Walker malformation (DWM). DWM is present in 6.8 in every 100 thousand live births, 13 and its main characteristics consist of cystic dilatation of the fourth ventricle and hypoplasia or agenesis of the cerebellar vermis ( Figure 1 ). 14 The condition presents with macrocephaly, hydrocephaly, developmental delay and/or ataxia. 14 Specifically, 80% of DWM patients present with hydrocephalus which frequently manifests with intracranial hypertension, macrocephaly, downward deviation of the eyeballs with retraction of the upper eyelids (setting sun sign), bulging of the fontanelle, dilation and congestion of the scalp veins and upward vertical gate paralysis. 15 Furthermore, ocular findings are common among DWS patients and also include lateral gaze palsy, nystagmus and strabismus, frequently presenting as diplopia. 16 Up to 40% of patient present with motor alternations including spasticity and around 20% with ataxia. 16

The etiopathogenesis of DWM is not completely clear. It is believed to be related to both genetic and environmental causes. 17 Particularly, in a study by Sun et al *.* chromosomal abnormalities were found in 32.9% of patients, whereas other authors have identified subchromossomal alterations, including *FOXC1* and *ZIC1/4* deletion. 17 18 19 Pre-natal intracerebral hemorrhage has also been associated with DWM. 20 Furthermore, animal evidence suggests that the etiology of DWM is associated with disturbed molecular signaling and, therefore, abnormal migration of RL and VZ derived cells, leading to vermian hypoplasia. 19 21

Another important group of cerebellar malformations consists of Joubert Syndrome and related disorders (JSRD), which have an incidence of 1 in 80 thousand live births 22 and are characterized by agenesis or hypoplasia of the cerebellar vermis, thickened superior cerebellar peduncles, and an enlarged interpeduncular fossa, resulting in a distinctive molar tooth sign on axial magnetic resonance imaging (MRI) or computed tomography (CT) scans ( Figure 1 ). 23 Clinically wise, JSRD presents with a combination of hypotonia, ataxia, developmental delay, intellectual disability, abnormal eye movements and breathing abnormalities. Particularly, JSRD patients frequently present with neonatal hypotonia which, when associated with irregular breathing pattern and altered eye movements, should suggest the diagnosis of JSRD. 24 Around 95% of patients present with global developmental delay and up ataxia is a common finding as well. 24 25 Joubert Syndrome and related disorders have been associated with several genetic variants related to cilia formation and function. 26 Moreover, previous studies have suggested that JSRD are associate with impaired granule cell proliferation in the RL, suggesting alterations in important molecular signaling pathways. 27 28

Cerebellar hypoplasia (CH) is another important group of cerebellar malformations, characterized by a reduction in the overall size of the cerebellum. It can manifest in various forms, including unilateral (affecting only one side), global (affecting the entire cerebellum), vermian (affecting the vermis), or pontocerebellar (affecting both the cerebellum and the pons) ( Figure 1 ). Ponto-cerebellar hypoplasia likely arises from the shared developmental origin of granule cells in the cerebellum and neurons in the pontine nuclei, both of which originate in the RL. 29 30 Patients frequently present with cognitive and motor alterations. In detail, up to 87% of patients present with developmental delay, which frequently include speech alterations, hypotonia and ataxia in around 50%, ocular findings including nystagmus, strabismus, or abnormal ocular movements in 40%, spasticity in 30 and seizures in up to 20%. 31

With regards to the etiology of CH, it is also associated with genetic and environmental factors. Several genes, including *CASK* , *DAB1* , *OPHN1* , *RELN* , *CHD7* , various tubulin genes, and several *TSEN* genes, have been linked to CH. 11 These genes play crucial roles in different stages of cerebellar development, such as progenitor cell proliferation, neuronal migration, and cell survival. Disruptions in these genes can lead to developmental defects and ultimately CH. In addition to genetic causes, CH can also result from non-genetic factors like congenital cytomegalovirus infection and perinatal exposure to alcohol and drugs like cocaine. 11

In the extreme of the cerebellar malformations, there is cerebellar agenesis (CA), which is characterized by complete or near-complete agenesis of the cerebellum and is a rare malformation. 11 While individuals with CA may experience notable neurological deficits, especially in movement and speech, their overall functioning can be preserved. 32 Particularly, the symptoms overlap with those of CH neonatal hypotonia, delayed motor development, ataxia, oculomotor abnormalities and cognitive disfunctions 33 Regarding etiology, it has been associated with *Ptf1a* deletions. 34 Particularly, as previously described, *Ptf1a* is important in the VZ to the development of Purkinje cells and other GABAergic neurons. Without those neurons, RL cells do not have trophic support and are also lost. 10 Therefore, the result is cerebellar agenesis.

It is possible to comprehend how the intricate relationship between the VZ, RL, RP and r1, and its respective molecular markers, is important for proper cerebellar formation and how any disturbance in these complex relations can lead to cerebellar malformation and dysfunction.

## MICROANATOMY AND CIRCUITRY OF THE CEREBELLUM

Despite representing only 10% of the brain's weight, the cerebellum contains up to 80% of the brain's neurons. 35 This discrepancy can be justified by its tightly folded structure, consisting of an inner white matter core covered by gray matter. This organization enables the cerebellum to have a unique and complex circuitry both within itself and in its connections to other parts of central nervous system. 36

**Figure 2:**
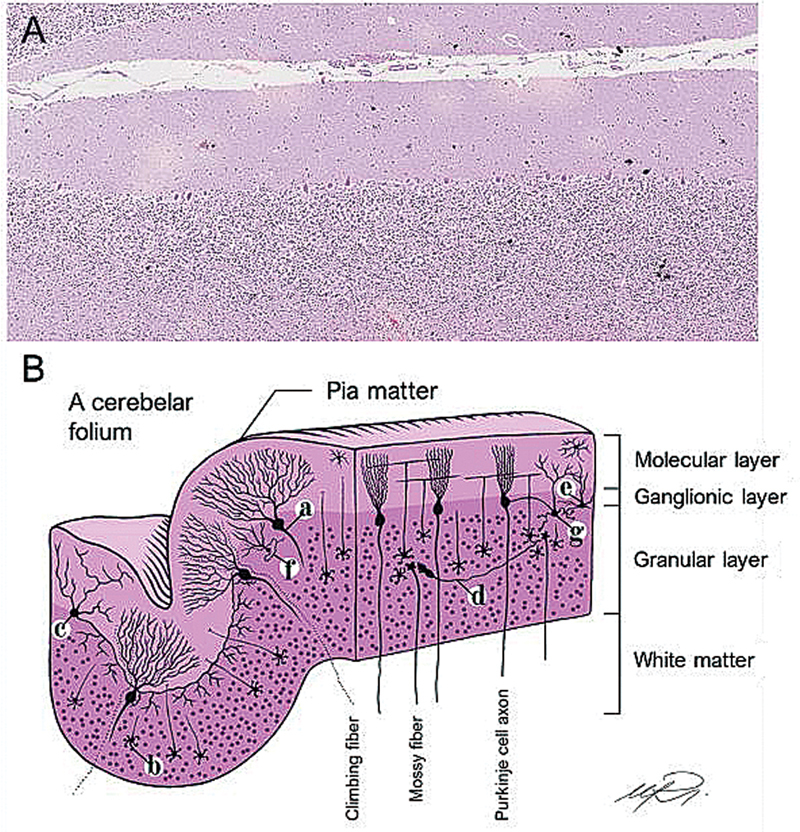
Microanatomy of the cerebellum. ( **A** ) Hematoxylin eosin staining showing the cerebellar microarchitecture in a panoramic view. ( **B** ) Graphical representation of the cerebellar cortex.

The cerebellar gray matter is organized into three layers: the internal granular layer, the middle Purkinje cell layer, and the external molecular layer ( Figure 2 ). The internal granular layer has cell bodies, Golgi cells, Lugaro cells, unipolar brush cells and Purkinje cell axons and consists of the input layer for the cerebellar cortex. 35 The middle layer is predominantly composed of Purkinje and Bergmann cells and performs the function of the output layer. 35 Finally, the outmost layer, the molecular layer, consists of stellate cells, basket cells, granule cells axons and Purkinje cells dendrites. 37 While Purkinje, Golgi, stellate and basket cells tends to be inhibitory, the granule and unipolar brush cells are excitatory. 37

**Figure 3:**
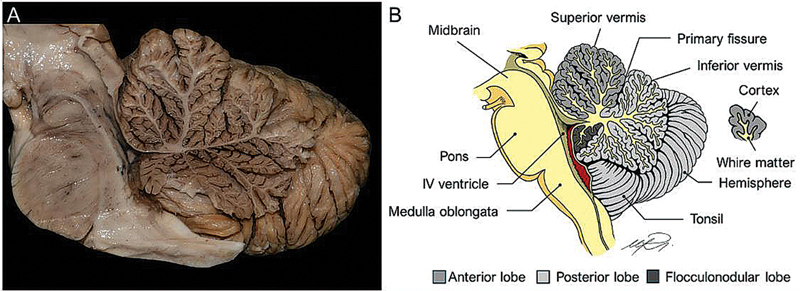
Macroanatomy of the cerebellum, sagittal section. ( **A** ) Sagittal dissection of the cerebellum and brainstem. ( **B** ) Graphical representation of the cerebellum in a sagittal section.

**Figure 4:**
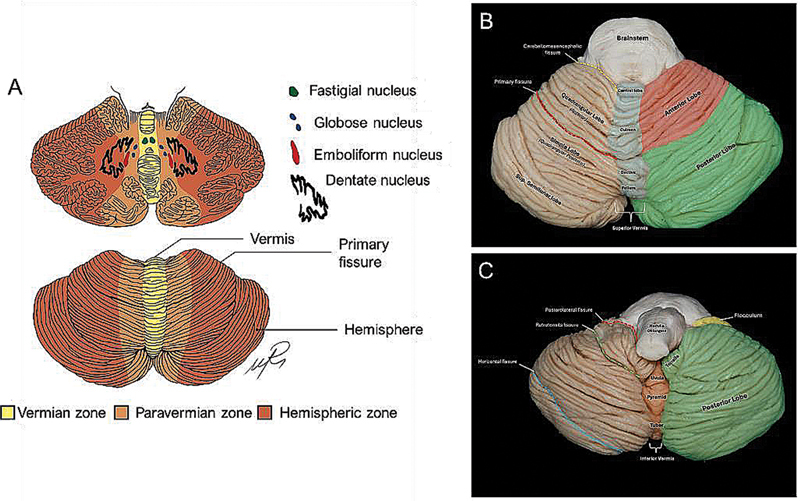
Macroanatomy of the cerebellum and cerebellar nuclei. ( **A** ) Graphical representation of the zones of the cerebellum and cerebellar nuclei. ( **B** ) Macroscopic appearance of the cerebellum.

The highly ramified white matter of the cerebellum resembles a tree in a sagittal section, an association first described by the Danish anatomist Benigmus Winslow in 1740, who named it *arbor vitae* (Latin for “tree of life”) ( Figure 3 ). This structure is embedded by three pairs of deep cerebellar nuclei: fastigial, interposed (consisting of globose and emboliform nuclei) and dentate nuclei, the largest one ( Figure 4 ). 35 All outputs from the cerebellum originate from those nuclei. 38

**Figure 5:**
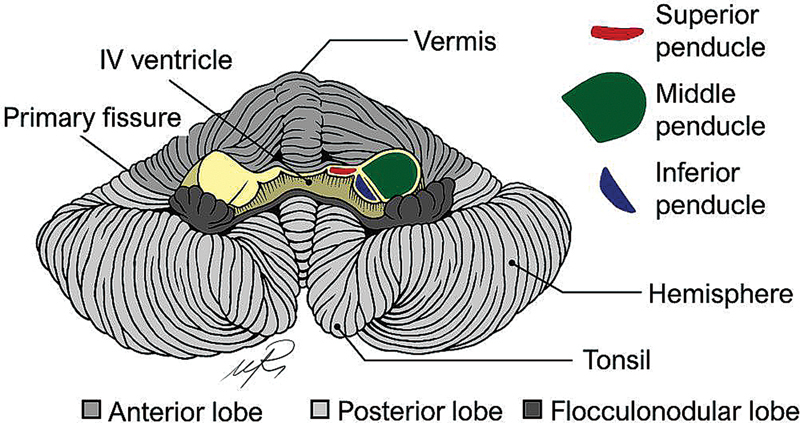
Graphical representation of the cerebellum and its peduncles.

The cerebellar peduncles play a major role connecting the cerebellum to other organs. Afferent pathways to the cerebellum are primarily transmitted through the inferior and middle cerebellar peduncles, while efferent pathways are conveyed through the superior cerebellar peduncle ( Figure 5 ). 39 40 Regarding the afferent input, the mossy and climbing fibers constitute the majority of cerebellar input. The former originate from pontine nuclei, spinal cord, brainstem reticular formation and vestibular nuclei and the latter originates exclusively in the inferior olive, both making excitatory projections. On the other hand, Purkinje cells are the sole source of output from the cerebellar cortex. 35 36 38 40

Functionally, the cerebellum can be divided in cerebro-, spino-, and vestibulocerebellum. The cerebrocerebellum is the largest division consisting mainly of lateral portions of the cerebellar hemispheres. It receives input from the cerebral cortex information via pontine nuclei and sends it to the thalamus and red nucleus. The spinocerebellum is formed by the anterior lobe, vermis and fastigial and interposed nuclei. It receives proprioceptive information from the dorsal column of the spinal cord and sends processed information back to adjust fine-tune movements. The vestibulocerebellum includes the flocculonodular lobe and paravermis. It receives inputs from vestibular nuclei and sends that information to both vestibular nuclei and the spinal cord to maintain trunk stability and balance. 35 36 37 38 40

## MACROANATOMY OF THE CEREBELLUM

The macroscopic organization of the cerebellum is essential for its proper function. In that sense, knowledge of its basic anatomy is of utmost importance to the practicing neurologist.

The cerebellum is located posterior to the brainstem and the fourth ventricle and is separated from the cerebrum by the tentorium ( Figure 3 ). Macroscopically, it can be divided into two hemispheres with a midline vermis ( Figure 4 ). Superficially, it presents several thin transverse folds named *folia* . 35 Each hemisphere can be divided into three lobes by two transverse fissures. The primary fissure separates the anterior from the posterior lobes, whereas the posterolateral fissure separates the posterior and flocculonodular lobes. 35 The cerebellum can be further divided by several fissures into 10 lobules using the Schmahmann classification. 41 42 Particularly, this classification arranges the lobules in numerical order, from I to X, radially in the sagittal plane. 41 For each lobule there is a central portion, in the vermis, and two lateral portions, in each hemisphere. 35

There are three pairs of white matter tracts that connect the cerebellum to the brainstem. 35 These structures, named cerebellar peduncles, contain cerebellar afferent and efferent tracts. 43 The middle cerebellar peduncle is composed mostly of pontocerebellar fibers that connect the contralateral pontine nuclei to cerebellar structures. 44 Medially to the middle cerebellar peduncle is the inferior cerebellar peduncle which contains afferent and efferent fibers. 35 As for the superior cerebellar peduncles, it is mainly composed of efferent pathways which originate from the dentate, interposed and fastigial nucleus, but also receives input from the ventral spinocerebellar tract. 45

## CLINICAL CORRELATES OF THE CEREBELLUM ANATOMY

Regarding function, the cerebellum has roles associated with motor functions, language, working memory, executive function, autonomic function and affect. 42 The basic knowledge of the cerebellar anatomy and function allow for an adequate understanding of its lesions and associated clinical manifestations. In that sense, a hallmark of cerebellar lesions are the movement alterations, which include dysmetria, dysdiachokinesia, postural and kinetic tremor as well as decomposition of movement. 46 Specifically, action tremor is usually associated with ipsilateral anterior lobe alterations, whereas hypermetria and decomposition of multi-joint movements are frequently associated with alterations in the dentate nucleus. 46 Moreover, it is possible to notice a somatotopy of the superior cerebellar cortex in the sense that upper limb ataxia is associated with lesions of the lobules IV, V, and VI while lower limb ataxia is associated with lesions in lobule III and IV. 46 Furthermore, the Guillain-Mollaret triangle, which is composed of the dentate-rubro-olivary pathway has an important role in the genesis of various types of tremors. 46

Particularly, concerning motor function, a complex circuitry is related to optimal movement execution. In that sense the cerebellum receives the motor planning and sensory proprioceptive information from its afferent pathways. This enables the cerebellum to compare the intended movement with what is being actually executed and perform the necessary corrections through its polysynaptic connections to the motor cortex. 47 The cerebellar areas involved in this complex movement orchestration, identified with functional magnetic resonance imaging in humans, include lobules I to VI and VIII. 47

**Figure 6:**
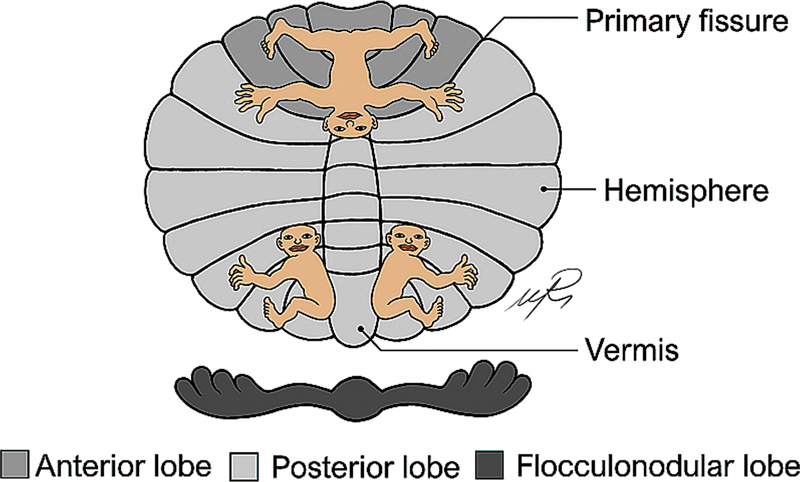
Graphical representation of the cerebellar homunculus.

Still considering movement and coordination alterations, it is important to mention the cerebellar homunculus ( Figure 6 ). Specifically, the cerebellum displays a particular somatotopic organization, similar to that of the motor and sensory cerebral cortices. 48 Furthermore, this somatotopic organization can be found clearly in the anterior lobe and, less evidently, in the posterior lobe. 48 49 In that sense, in the anterior lobe, it is possible to notice a clear anterior-to-posterior somatotopic organization going from representation of the feet to the hands and the head. 48 50 On the other hand, on the posterior lobe, several representations of body parts can be identified; however, the general organization varies between patients and studies. 48 49 Furthermore, there is evidence that the anterior somatotopic map responds to both ipsilateral and contralateral activity, whereas the anterior representation responds only to ipsilateral movement. In general, the presence of a cerebellar homunculus further highlights the importance of the clinic-anatomic relations.

Other classic alterations of cerebellar function are related to gait and posture. In that sense, patients with cerebellar lesions might present with broad-based stance and gait, as well as difficult standing up. 46 Alterations in gait can be seen in alterations of the fastigial and interposed nuclei. In that sense, previous studies have identified a correlation between posture/gait scores and medial/intermedial cerebellar volume. 51 Furthermore, lesions in the posterior paravermis, nodulus and culmen can present with lateropulsion. Another manifestation of cerebellar dysfunction include decomposition. It is frequently associated with dentate nuclei lesion, which also present with hypermetria as well as lateral and intermediate parts of the cerebellum. 46

Moreover, the alterations of ocular movements that are associated with cerebellar lesion encompass instability of gaze and nystagmus, hypermetria/hypometria of saccades, saccadic pursuit, skew deviation and disorders of vestibulo-ocular reflex as well as optokinetic responses. 46 The cerebellar structures which are necessary for adequate ocular movements include the lobules VI, VII, crus I and II of ansiform lobule, flocculus, paraflocculus, uvula and nodulus. Particularly, a nucleus of great importance for oculomotor function is the fastigial nucleus, which is important for head orientation, eye-head gaze shifts, saccades and smooth pursuit. 46 Previous studies have identified that reactive oculomotor functions are mostly related to medial cerebellar lesions, whereas voluntary action is affected in lateral lesions. 46

Cerebellar lesions can also lead to speech alterations. 46 In that sense, in patients presenting with speech deficits due to cerebellar alterations, damage of the paravermal region, hemispheric lobules I and VI and dentate nucleus are commonly identified. 46 Previous research indicated that lesions on the left cerebellum would be more associated with language alterations; however, recent findings show that lesions of both sides may cause speech alterations. 46 Moreover, two networks seem to be associated with speech control:

- the sensory motor cortex, the basal ganglia, thalamus and inferior cerebellum and;
- the supplementary motor area, the dorsolateral frontal cortex, the anterior insula and the superior cerebellum. 52

It is also important to notice that there is plentiful evidence pointing to the importance of the cerebellum in cognition. 53 The range of cognitive skills in which the cerebellum seems to play a role include verb generation, verbal working memory, verbal fluency, visuospatial tasks, executive functions, emotion, pain and addiction. 46 54 55 However, clinical signs of cognitive dysfunction associated with cerebellar lesion are usually subtle and mostly noticeable in acute settings or congenital disorders. 46 Regarding topography, previous studies have identified that right posterolateral cerebellar hemispheric lesions are associated with dysfunctions in verbal fluency, that paravermal lesions are associated with lower performance in total aphasia score and that bigger cerebellar lesions are associated with worse reading function when compared to smaller focal lesions. 55 56 Moreover, extensive vermal damage has been associated with affective symptoms and personality changes, which include anxiety, aggression as well as hyperspontaneous and disinhibited behavior. 46 56 57 58 In that sense, affective symptoms and personality changes are observed after vermal damage in children being subjected to cerebellar tumor resection. The vermis is connected to the limbic system, and its stimulation might modulate amygdala activity, which would suggest a pathway associated with emotional responsiveness. 46

## VASCULAR ANATOMY OF THE CEREBELLUM

**Figure 7:**
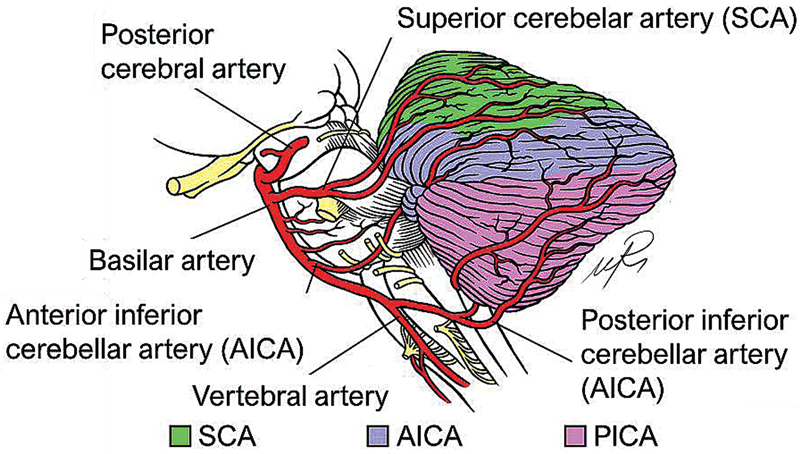
Graphical representation of the cerebellar vascular territories.

**Figure 8:**
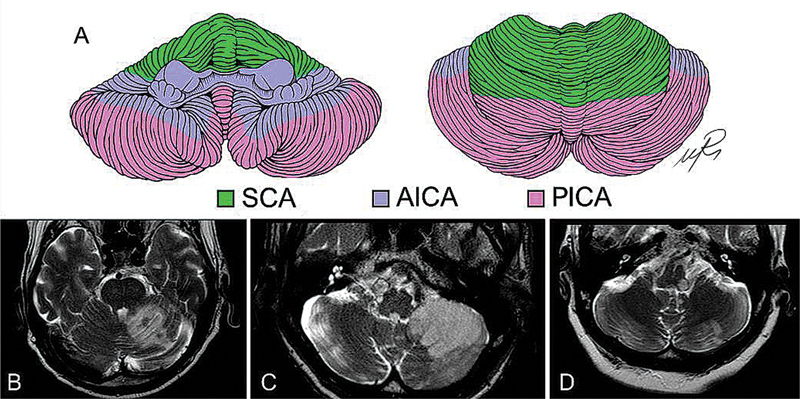
Abbreviations: AICA, anterior inferior cerebellar artery; FLAIR, fluid-attenuated inversion recovery; SCA, superior cerebellar artery; PICA, posterior inferior cerebellar artery. Cerebellar strokes. ( **A** ) cerebellar vascular territory. ( **B–D** ) MRI and FLAIR scans showing SCA, AICA and PICA strokes respectively. ( **C** ) Dorsolateral bulbar lesions compatible with Wallenberg syndrome.

Regarding the arterial supply of the cerebellum, there are three main arteries: the superior cerebellar artery (SCA), the anterior inferior cerebellar artery (AICA) and the posterior inferior cerebellar artery (PICA) ( Figure 7 ). With regards to the SCA, it is the most constant of the cerebellar arteries and usually arises from the basilar artery or the first segment of the posterior cerebral artery. 59 The SCA usually supplies the rostral portion of the cerebellar hemispheres and vermis, as well as part of the white matter and dentate nucleus, as well as some portions of the brainstem ( Figure 8 ). 60 Patients presenting with SCA infarct can be divided into two groups. In the first one, patients present with unilateral ataxia and brainstem dysfunction due to proximal SCA occlusion whereas, in the second, patients present exclusively with cerebellar signs, due to distal occlusion of the vessel. 61 In general, most common symptoms associated with SCA infarction include vertigo (60%), dysarthria (55%), nystagmus (50%), limb ataxia (48%), gait ataxia (45%) and dysmetria (43%). 62 Particularly, dysarthria is frequently associated with signs of infarction of the medial branch of the superior cerebellar artery. 46

As for the AICA, it frequently arises from the lower third of the basilar trunk. 59 It is responsible for the irrigation of the anteroinferior region of the cerebellum, which includes the flocculus ( Figure 8 ). It also supplies the middle cerebellar peduncle, the lateral portion of the pons and the inner ear. 60 However, it is important to notice there is important interpatient variability regarding the territory of the AICA. 59 Most common symptoms associated with AICA ischemia include vertigo, impaired hearing, gait ataxia, limb ataxia, nystagmus, diplopia and peripheral nerve palsy. 63 64

With regards to the PICA, it usually emerges from the vertebral arteries. 59 However, it can arise from a common trunk with the AICA from the basilar artery, from the extradural vertebral artery, from the ascending pharyngeal artery or from other origins. 59 As for the territory of irrigation of the PICA, it involves the caudal part of the cerebellar hemispheres and vermis ( Figure 8 ). 65 It is also responsible for the irrigation of the dorsolateral region of the medulla oblongata. 60 It is important to notice that there is a reciprocal relation between the territories of the PICA and AICA. 60 In that sense, if the PICA is hypoplastic, the AICA assumes a larger territory. 60 Infarcts of the PICA usually manifest with vertigo, trunk and limb ataxia, nystagmus, dysmetria, and lateropulsion. 60 Particularly, PICA ischemia is usually more correlated with oculomotor signs than other cerebellar arteries infarction. 46 A classical manifestation of PICA ischemia is the lateral medullary syndrome, also known as Wallenberg syndrome. 66 Manifestation might include vertigo with nystagmus, nausea, vomiting, dysphagia, dysphonia, dysarthria, hiccup, ipsilateral ataxia, ipsilateral Horner syndrome, and impairment of pain and thermal sensation on the ipsilateral face and contralateral body. 66

With regards to the venous drainage of the cerebellum, that are the groups of veins: the anterior, the posterior, and the superior. 67 68 69 The anterior, or petrous, group drains the anterior portion of the cerebellum into the superior and inferior petrous sinus. It consists of the anterior pontomesencefalic vein, the petrosal vein and the lateral mesencephalic vein. 69 The posterior group, also known as the tentorial group, includes the veins draining to the tentorium and is composed by the inferior vermian veins and its tributaries. 69 Finally, the superior, or vein of Galen, group drains the upper part of the cerebellar hemispheres, vermis and midbrain to the vein of Galen and the straight sinus. 69 These group consist of precentral cerebellar, superior vermian, and posterior mesencephalic vein. 69

## RADIOLOGICAL ASPECTS OF THE CEREBELLUM

Neuroimaging plays a pivotal part in the evaluation of the cerebellum and its associated pathologies, offering critical insights for the diagnosis, prognosis and understanding of clinical manifestation in cerebellar disorders.

Traditional imaging modalities such as prenatal and postnatal ultrasound, as well as MRI, are particularly effective in detecting early developmental anomalies, such as cerebellar hypoplasia, and malformations, including Dandy-Walker syndrome and Arnold-Chiari malformation. 70 Conventional MRI sequences—such as T1, T2, fluid-attenuated inversion recovery (FLAIR), short Tau inversion recovery (STIR), diffusion, susceptibility-weighted imaging (SWI), and magnetic resonance (MR) angiography—are effective not only for delineating the topography of cerebellar deficits but also for suggesting the underlying etiology, which may include degenerative, inflammatory, infectious, or metabolic causes. 70 71

Magnetic resonance imaging is highly useful in identifying focal cerebellar lesions (e.g., infarcts, tumor, demyelinating plaques), cerebellar atrophy patterns and metabolic disorders that preferentially affect the cerebellum. Regarding radiological-clinical correlation, for example, imaging that reveals anterior lobe atrophy can correlate with gait ataxia and lower limbs disfunction, whereas lesions in lobules VI and VII may be associate with oculomotor impairment and cognitive dysfunction. 72

Moreover, functional imaging techniques, including single-photon emission CT (SPECT), positron emission tomography (PET), and functional MRI (fMRI), are enhancing our understanding of the complex cerebellar circuitry. These advances have been particularly useful in studying conditions such as the cerebellar cognitive affective syndrome. 73

Considering that fMRI can be used to evaluate cerebellar activation patterns during motor, cognitive, or emotional task, studies have demonstrated that lobules VI, crus I and crus II are directly involved in cognitive functions such as working memory and language. Furthermore, resting-state fMRI has underscored cerebellar involvement in complex brain networks, including the default mode network and salience network, as well as its functional connectivity with the prefrontal cortex, parietal cortex and limbic system. 72

On the other hand, PET and SPECT have been used to investigate cerebellar perfusion and metabolic activity in degenerative ataxias, cerebellar cognitive affective syndrome and cognitive-psychiatric disorders. For instance, hypometabolism in the posterior cerebellar hemisphere may be associated to language deficit and executive dysfunction. 74

In conclusion, as a central hub for integrating sensory and motor information, the cerebellum is a structure that plays a central role in coordinating movement, maintaining postural stability, and supporting cognitive functions. Its intricate development from the embryonic stage, as well as its complex microanatomy, circuitry, microanatomy, and vascular supply highlight the importance of the continuous study of these subjects.

Moreover, a full comprehension of this remarkable structure is essential for adequate understanding of the diseases associated with its dysfunction and the profound clinical manifestations associated with it.
